# Factors That Influence Perceived Organizational Support for Emotional Labor of Chinese Medical Personnel in Hubei

**DOI:** 10.3389/fpsyg.2021.684830

**Published:** 2021-06-10

**Authors:** Zhi Zeng, Xiaoyu Wang, Haoran Bi, Yawen Li, Songhua Yue, Simeng Gu, Gaoyue Xiang

**Affiliations:** ^1^School of Health Economics and Management, Nanjing University of Chinese Medicine, Nanjing, China; ^2^School of Humanities and Social Science, Nanjing Institute of Technology, Nanjing, China; ^3^Department of Psychology, Jiangsu University Medical School, Zhenjiang, China; ^4^Organization Department, The First Affiliated Hospital of Soochow University, Suzhou, China

**Keywords:** organizational support, emotional labor, professional identity, medical personnel, fight against COVID-19

## Abstract

At the outbreak of coronavirus disease in Wuhan, China, 42,322 medical personnel from other provinces and municipalities in China volunteered to rush to Hubei to assist their colleagues. Their all-out efforts contributed to Hubei finally winning the fight to prevent and control the pandemic. The aim of this study is to explore the influence of perceived organizational support on the emotional labor of medical personnel in Hubei Province. A group of 170 medical personnel from (tertiary) hospitals who participated in the pandemic aid operation in Hubei completed self-administered questionnaires, including the perceived organizational support scale, emotional labor scale, and professional identity scale. This study used Pearson's correlation in SPSS to analyze the three variables of organizational support, emotional labor, and professional identity. Organizational support and emotional labor (*r* = 0.443, *P* < 0.01), organizational support and professional identity (*r* = 0.631, *P* < 0.01), and emotional labor and occupational identity (*r* = 0.511, *P* < 0.01) showed a significant positive correlation. The bootstrapping mediating effect test was used to determine the overall mediating effect of occupational identity. Occupational identity was a complete mediating effect between organizational support and emotional labor. The results show that a strong sense of organizational support can promote higher emotional labor among medical workers in Hubei Province. A strong sense of organizational support will also promote a stronger professional identity; further, a strong professional identity completely mediates the effect of perceived organizational support on emotional labor. These results infer that in emergency medical and health services, medical personnel can realize a high sense of organizational support, which could enhance their professional identity; this enables them to combine their professional goals with organizational goals more actively and to finally pay higher emotional labor to achieve organizational goals.

## Introduction

To win the battle against coronavirus disease (COVID-19), 42,322 medical workers from various provinces and cities in China went to Hubei Province to support their colleagues. When treating COVID-19 patients (especially critically ill patients), doctors and nurses rely on their beliefs and empathy to curb their own anxiety and fear; when faced with dying patients with severe infection, medical personnel must regulate their own emotions to accept the limitations of treatments and avoid excessive empathy, which may impact their performance in the battle against the disease. These circumstances require medical workers who are highly professional and who are able to work in these circumstances while investing appropriate emotional labor.

Existing studies show that emotional labor is the manner in which medical personnel express emotion according to the established requirements of the organization or the emotional expression of the organization's needs (Ashforth and Humphrey, 1993). In this type of medical emergency, the organization regulating the emotional labor of medical personnel is significant for both the patients and the well-being of the organization (Henderson and Borry, 2020).

Therefore, we can infer that when the medical personnel in Hubei feel trust and support from their organization, they will experience a sense of purpose and be more willing to accept the high-intensity work overload and invest higher emotional labor in the frontlines of the fight against the COVID-19 pandemic. This study uses organizational identity as a mediator to explore the mechanism of the impact of perceived organizational support on the emotional labor of medical personnel, which has both theoretical value and practical significance. Against the current social backdrop of the COVID-19 pandemic, this study innovatively considers the emotional labor of medical personnel from the perspective of organizational support. Our findings enrich existing theoretical research on medical personnel's emotional labor. It also provides practical constructive outcomes, such as possible countermeasures against the negative aspects of investing emotional labor, for hospital management to implement from the perspective of human resources management.

## Theoretical Basis and Research Hypothesis

### Perceived Organization Support and Emotional Labor

Based on the social exchange theory and the organizational support theory, Eisenberger, an American social psychologist specializing in emotional labor, put forward the concept of organizational support according to the principle of reciprocity. This work lays the foundation for future studies on organizational support (Eisenberger et al., 1986; MacMillan, 1997). and Rhoades and Eisenberger (2002) continued to enrich and update the concept of organizational support. Chinese scholar Ling et al. (2006) and others first put forward that the concept of organizational support in China differs from that of foreign countries. They posit that in a Chinese environment, employees perceive organizational support as support in their work, concern for their interests, and recognition of their values. This concept has been well-supported and verified in the study of organizational support in China.

During the past 10 years, scholars in other countries went beyond superficial research; studies gradually started showing organizational support to be a mediating variable and an adjusting variable. Further, the relationship between the two factors have been investigated to determine the mechanism and model construction of the sense of organizational support as a whole; thus, the professional status of medical personnel has been comprehensively analyzed according to the model (Galletta et al., 2011; Labrague et al., 2018; Yang et al., 2019; Poghosyan et al., 2020). Based on this research, corresponding countermeasures and suggestions to improve employees' sense of organizational support, reduce turnover intention, and manage emotional labor were put forward. Research shows that good social support can enhance self-awareness, reduce psychological stress reactions, and moderate the negative impact of stressful events (Li et al., 2021).

During his research on airline flight attendants, American social psychologist (Russell, 1983) proposed the concept of emotional labor for the first time. He posits that emotional labor is the third form of labor, after physical and mental labor. It can be said that Hochschild's work pioneered the current research area of emotional labor. Scholars from different countries have investigated emotional labor based on different service industries, including both qualitative research and empirical research (Ashforth and Humphrey, 1993; Grandey, 2000; Delgado et al., 2017; Zhao and Xi, 2017).

A literature review shows that qualitative research generally analyzes the connotation, theory, and strategy of emotional labor, while empirical research mainly focuses on the service industry, teachers, medical personnel, and other professions (Glomb and Tews, 2004; Larson and Yao, 2005; Back et al., 2020; Wang et al., 2020). It has been found that the focus of research on medical personnel's emotional labor by scholars globally has gradually shifted from the adverse effects of medical personnel's emotional labor (work pressure, job burnout, etc.) to developing methods for stabilizing the medical team, improving the quality of medical service (reducing the turnover rate, improving job satisfaction, etc.), formulating emotional labor management strategies, and improving the current situation of medical service quality.

Considering previous scholars' theoretical research on organizational support and emotional labor, we observe a close relationship between organizational support and emotional labor; further, considering whether organizational support has a negative impact on emotional labor, we propose the following hypotheses:

Hypothesis H1: The sense of organizational support can positively predict emotional labor among medical personnel in Hubei Province.

Hypothesis H2: The sense of organizational support has a positive effect on the emotional labor of medical personnel in Hubei Province.

### Organizational Support and Professional Identity

Based on Erikson's ego identity theory, early scholars in various industries have proposed their own views on professional identity (Zhou and Guo, 2006). Some scholars suggest that professional identity is more of an emotional construct, a desire to stay in the current occupation, and a degree of love for the existing occupation (Blau, 1988; Moore and Hofman, 1998). With the supplementing and improvement of the concept of professional identity by other scholars, researchers gradually started investigating professional identity in each industry. Some researchers also started conducting in-depth research on professional identity in various industries. The professional identity of medical industry personnel were included in the construction of a model and used to study the interaction between professional identity and other factors or as an intermediary (Selma and Selma, 2015; Kyratsis et al., 2017; Matthews et al., 2020). Previous studies found that professional identity is influenced by perceived organizational support. This means that, if the medical personnel in Hubei Province have a higher sense of organizational support, they will identify with their work more and put more energy into medical treatment. Therefore, we propose the following hypothesis:

Hypothesis H3: Perceived organizational support has a positive impact on the professional identity of medical personnel in Hubei Province.

### Emotional Labor and Professional Identity

In the process of medical treatment, the emotional labor of medical personnel can be expressed in diverse ways, such as hiding real emotions, changing emotions to adapt to the requirements of the organization, or naturally showing and revealing emotions. Di Monte et al. (2020), Di Trani et al. (2021), and their research teams conducted in-depth research on the relationship between job burnout and various psychological characteristics of Italian medical personnel during the COVID-19 emergency. They found that when relevant conditions at the organizational level are difficult to control, medical personnel can help prevent job burnout caused by emotional labor by enhancing individual resources such as personal skills training and self-psychological support. This is especially true for medical personnel who came to aid Hubei during the pandemic. However, the degree and performance of emotional labor are affected by various factors. Scholars from all walks of life have launched a fierce discussions on the influencing factors of emotional labor. Here, empirical research investigating the impact of professional identity on emotional labor attracted wide attention (Zeng and Shen, 2013; Forouzadeh et al., 2018; Willetts and Garvey, 2020). Research shows that professional identity also has a positive effect on emotional labor; that is, when medical personnel have high professional identity, they are willing to invest more emotional labor to meet organizational and professional requirements. We therefore propose the following hypothesis:

H4: Professional identity has a positive impact on emotional labor.

### The Intermediary Effect of Professional Identity

Although few empirical studies have directly explored the relationship between perceived organizational support, professional identity, and emotional labor, some related studies focused on the internal connection among these three variables. Accordingly, this study introduces professional identity as a variable. We believe that the influence of the emotional labor invested by medical personnel while aiding Hubei is realized through the intermediary variable of professional identity. The organizational support experienced by medical personnel has an impact on their professional identity first; then, professional identity affects the emotional labor invested by medical personnel in the fight against the pandemic. We therefore propose the following hypothesis:

H5: The effect of perceived organizational support on emotional labor among medical workers in Hubei Province is mediated by professional identity.

**Figure 1:**
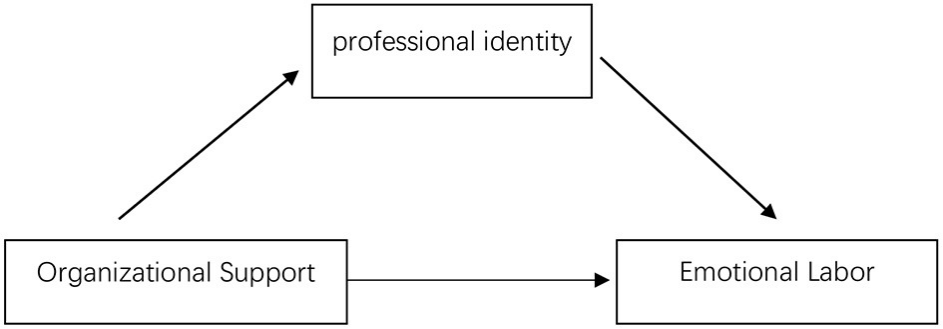
Theoretical model of this study.

Based on these theories and hypotheses, this study can be summarized as a theoretical model that includes intermediary links, as shown in Figure 1.

## Research Design

### Research Objects

This study adopted a convenience sampling method. The respondents were medical personnel from third tier (tertiary) hospitals in Beijing, Jiangsu, Shanghai, Hunan, Guangdong, Sichuan, and other towns or cities who participated in the pandemic aid operation in Hubei. Electronic questionnaires were distributed to respondents and each questionnaire included an agreement of informed written consent. A total of 170 questionnaires were distributed and all 170 valid questionnaires were returned, a response rate of 100%. There were no missing answers in the questionnaires and no repetitive answers. Written informed consent was obtained before the experiments, and the study was approved by the committee of the ethnic board of Nanjing University of Chinese Medicine and the latest revision of the Declaration of Helsinki.

### Research Tools

#### Perceived Organizational Support Scale

This study adopted the perceived organizational support scale (Ling et al., 2006) and used a 6-point Likert scale ranging from 1 = “Strongly oppose” to 6 = “strongly approve.” Based on the pre-survey reliability and validity analysis results, 20 items and three dimensions were retained and four items were eliminated. These items were eliminated as they influenced the validity and reliability of the scale negatively; this applies to all other items removed from the research tools. The Cronbach's α coefficient of the scale was 0.968, indicating acceptable reliability.

#### Emotional Labor Scale

We adopted the emotional labor scale (Diefendorff et al., 2011) and used a five-point Likert scale ranging from 1 = “Never” to 5 = “Always” for the answers. Based on the pre-survey analysis results, nine items and three dimensions were retained after eliminating five items. The Cronbach's α coefficient of the emotional labor scale was 0.852.

#### Professional Identity Scale

We used the nurse's professional identity scale, translated and verified by Hong et al. (2010). We used a five-point Likert scale ranging from 1 = “Never” to 5 = “Completely.” Based on the pre-survey and analysis results, one question was excluded and 20 items in seven dimensions were retained. The Cronbach's α coefficient of the professional identity scale was 0.934.

### Procedure and Analysis

The three scales described above were either from a questionnaire developed by foreign scholars or from a questionnaire developed for personnel in other industries as research targets; therefore, while ensuring the equivalence of item meaning, we made appropriate adjustments and corrections by combining the relevant items in the scales according to the professional characteristics of the participants and conducted a pilot survey before the formal survey. We then revised the relevant items of the questionnaire according to the feedback of the pilot survey. To ensure the quality of the questionnaires, we contacted the nursing departments and medical administration divisions of the relevant hospitals and asked them to distribute the electronic questionnaires to their personnel. We emphasized that the questionnaire information would only be used for research purposes and that personal information will be kept confidential. The participants were asked to complete the questionnaires according to their working conditions and the completed questionnaires were handed directly to the researchers, without any feedback to the hospital. Therefore, the questionnaire recovery rate in this study was 100%.

SPSS 23.0 software was used for descriptive statistics. We also used a Pearson's correlation analysis and a bootstrap test. The specific statistical analysis process was as follows: first, we used the pilot survey data to test the reliability and validity of the three research tools using a reliability analysis, validity analysis, and confirmatory factor analysis. Second, we conducted a descriptive statistical analysis and correlation analysis of the main research variables. Finally, we used an intermediary regression analysis and an Amos path analysis to investigate the mediating role of professional identity.

## Research Results

### Participants' Demographic Characteristics

The demographic characteristics of the medical personnel surveyed in this study are shown in Table 1. Of the sample, 48 (28.24%) were male medical personnel and 122 (71.76%) were female. The sample age was concentrated in the 31–40 years old range, accounting for 62.94%. Regarding marital status, most participants (83.53%) were married. Considering education level, 90% of the participants have a bachelor's degree or master's degree. The years of work experience were concentrated in the 6–10 and 11–15 years brackets, accounting for 29.41 and 34.71%, respectively. This indicates that most of the medical personnel who participated in the pandemic aid operation in Hubei were employees with a number of years' experience. From the perspective of the composition of the titles of medical personnel, those with intermediate titles accounted for half of the total. In terms of personnel structure, 77.65% of them were nurses, 18.82% were doctors, 1.18% were medical technicians, and 2.35% were administrative staff. The basic composition of the sample was shown to be consistent with that of the more than 40,000 medical workers who took part in the pandemic aid initiative in Hubei Province, according to the National Health Commission of China (China News Network, 2020). This indicates that the sample is representative and accurately reflects the medical personnel situation in Hubei Province.

**Table 1:** Demographic characteristics of the medical personnel.

**Demographic variables**	**Category**	**Frequency**	**Percentage (%)**
Gender	Male	48	28.24
	Female	122	71.76
Working years	5 years or less	12	7.06
	6–10 years	50	29.41
	11–15 years	59	34.71
	16–20 years	22	12.94
	More than 20 years	27	15.88
Professional level	Junior	37	21.76
	Intermediate	85	50
	Deputy high	40	23.53
	Senior	8	4.71

### Analysis of Differences Based on Demographic Variables

This study selected three demographic variables (gender, working years, professional level) to test whether they impact organizational support, emotional labor, and professional identity.

#### Analysis of Differences Based on Gender

To examine differences based on gender, this study conducted an independent sample *t*-test on the organizational support, emotional labor, and professional identity of medical personnel assisting Hubei. The test results are shown in Table 2.

**Table 2:** Results of the *t*-test of independent samples based on gender differences.

**Variables**	**Male (*n* = 48)**	**Female (*n* = 122)**	**T**
Organizational support	4.493 ± 0.916	4.475 ± 0.931	0.112
Emotional labor	3.833 ± 1.012	4.184 ± 0.870	−2.259^*^
Professional identity	4.266 ± 0.445	4.233 ± 0.501	0.404

* *p < 0.05*.

According to the statistical results, the differences based on gender for the two variables of organizational support and occupational identity were not significant. However, for emotional labor, the mean for men was 3.833, the mean for women was 4.412, the *T*-value was −2.259, and it was significant at the level of *P* < 0.05. This shows that women's emotional labor is significantly higher than men's, which may be due to differences in thinking, behavior, and empathy between the sexes.

#### Analysis of Differences Based on Working Years

To examine differences based on working years, this study conducted a one-way analysis of variance test on the organizational support, emotional labor, and professional identity of medical personnel assisting Hubei. The test results are shown in Table 3.

**Table 3:** Results of the one-way analysis of variance test based on working years.

**Variables**	**5 years or less** **(*n* = 12)**	**6–10 years** **(*n* = 50)**	**11–15 years** **(*n* = 59)**	**16–20 years** **(*n* = 22)**	**More than 20 years** **(*n* = 27)**	***F***
Organizational support	4.289 ± 0.956	4.528 ± 0.941	4.671 ± 0.886	4.214 ± 0.931	4.275 ± 0.914	1.605
Emotional labor	4.188 ± 0.749	4.243 ± 0.509	4.311 ± 0.625	4.085 ± 0.509	4.232 ± 0.555	0.994
Professional identity	4.417 ± 0.515	4.607 ± 0.470	4.627 ± 0.480	4.455 ± 0.520	4.840 ± 0.338	2.782^*^

* *p < 0.05*.

According to the statistical results, the differences based on working years for the two variables of organizational support and emotional labor were not significant. However, there were significant differences in occupational identity among medical personnel based on working years. Among the medical personnel assisting Hubei, generally, as the number of working years increased, there was a stronger recognition of their work and professional roles. This may be because as work experience increases, medical personnel deepen their understanding of their work and are more able to integrate into the job role, leading to a higher understanding of the professional role.

#### Analysis of Differences Based on Professional Level

To examine differences based on professional level, this study conducted a one-way analysis of variance test on the organizational support, emotional labor, and professional identity of medical personnel assisting Hubei. The test results are shown in Table 4.

**Table 4:** Results of the one-way analysis of variance based on professional title.

**Variables**	**Junior (*n* = 37)**	**Intermediate (*n* = 85)**	**Deputy high (*n* = 40)**	**Senior (*n* = 8)**	***F***
Organizational support	4.307 ± 0.859	4.652 ± 0.945	4.334 ± 0.904	4.184 ± 0.896	2.059
Emotional labor	4.128 ± 0.603	4.302 ± 0.542	4.125 ± 0.574	3.953 ± 0.750	1.796
Professional identity	4.138 ± 0.487	4.285 ± 0.495	4.255 ± 0.484	4.204 ± 0.359	0.819

According to the statistical results, there were no significant differences based on professional level for the three variables of organizational support, emotional labor and professional identity.

### Descriptive Statistical Analysis of Variables

The study involves three variables—perceived organizational support, emotional labor, and professional identity—and we focus on descriptive statistics to determine the overall performance of these variables. We conducted descriptive statistics analyses for each variable; the results are shown in Table 5. We see that the average value for organizational support is 4.4799, with a standard deviation of ±0.92402. The maximum value is 6, and the minimum value is 2; this indicates that although there are differences in the sense of organizational support among the medical personnel participants, their sense of organizational support is still strong. The average value for emotional labor is 4.2059 (±0.57747), indicating that the emotional labor level of medical personnel in Hubei Province was very high. The average value of professional identity was 4.2424 (±0.28286). This indicates that the professional identity of medical workers in Hubei Province was very high.

**Table 5:** Descriptive statistics of main variables.

**Variables**	**Average**	**Maximum**	**Minimum**	**Standard deviation**
Organizational support	4.4799	6.00	2.00	±0.92402
Emotional labor	4.2059	5.00	2.63	±0.57747
Professional identity	4.2424	5.00	2.53	±0.48486

### Analysis of the Correlation Between Variables

To determine the relationships among perceived organizational support, emotional labor, and professional identity, we must first determine whether correlations exist among the three variables. We utilized a Pearson's correlation analysis to analyze the three variables; the results are shown in Table 6.

**Table 6:** Pearson's correlation analysis of variables (*N* = 170).

	**Organizational support**	**Emotional labor**	**Professional identity**
Organizational support	1		
Emotional labor	0.443^**^	1	
Professional identity	0.631^**^	0.511^**^	1

** *p < 0.01*.

Table 6 shows that the relationships among the variables—perceived organizational support and emotional labor (*r* = 0.443, *p* < 0.01), perceived organizational support and professional identity (*r* = 0.631, *p* < 0.01), and emotional labor and professional identity (*r* = 0.511, *p* < 0.01)—were all significantly positively correlated. We can therefore state that the perceived organizational support, emotional labor, and professional identity of medical workers in Hubei Province are pairwise correlated.

### Mediating Effect of Professional Identity

The above empirical analysis verified the influence of perceived organizational support on emotional labor; these results confirm our hypotheses. In terms of the pandemic aid operation, perceived organizational support has a positive impact on emotional labor. Further, this conclusion applies equally to the causal relationship between perceived organizational support and professional identity and the causal relationship between emotional labor and professional identity. Next, we need to verify the mediating effect of professional identity on perceived organizational support and emotional labor. Considering that the test for mediating effect using the SPSS regression method may not be sufficient, this study uses the Amos path analysis method for further testing and obtains the final test results. First, the path test method is used to determine whether the path is significant; next, bootstrapping is used to observe whether the upper and lower limits contain zero.

**Figure 2:**
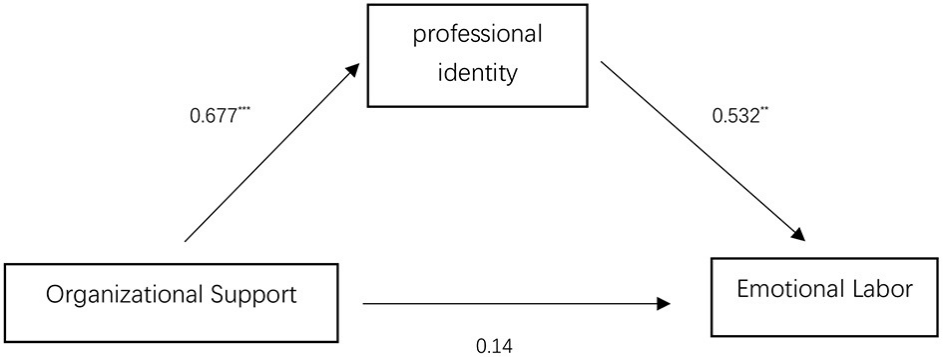
Mediating effect model of professional identity on the relationship between perceived organizational support and emotional labor.

The mediating effect of professional identity was tested and a model diagram was drawn (Figure 2). The results show that the X2 / DF is 4.104 < 5, which is acceptable. The fitting indexes are ~0.9 and the fitting is passed. The path test results showed that the critical value (composite reliability – CR) of organizational support for professional identity was CR = 7.872 > 1.96, the critical value of professional identity to emotional labor is CR = 2.808 > 1.96. The critical value of organizational support to emotional labor is CR = 1.147 < 1.96, which is not up to the standard. It was found that all factor load *p*-values were significantly lower than 0.01, except for the path between perceived organizational support and emotional labor, which was not significant (*p* = 0.252). This shows that perceived organizational support has a significant influence on professional identity, which, in turn, has a significant influence on the path of emotional labor.

Next, we used a bootstrapping mediating effect test to examine the mediating effect of overall professional identity (Table 7).

**Table 7:** Mediating effect of professional identity on perceived organizational support and emotional labor.

**Effect**	**Path**	**Path coefficient**	**95% confidence interval (CI)**
Indirect effect	Path 1	0.080	[0.029, 0.179]
Direct effect	Path 2	0.031	[−0.027, 0.112]
Total	Path 3	0.111	[0.038, 0.193]

*Path 1: Organizational Support → Professional Identity → Emotional Labor*.

*Path 2: Organizational Support → Emotional Labor*.

*Path 3: Path 1 + Path 2*.

The path coefficient of the indirect effect of professional identity on the relationship between perceived organizational support and emotional labor is 0.080, and the upper and lower limits of the confidence interval are 0.029 and 0.179, respectively. These are both positive and do not contain zero, indicating that there is an indirect effect. The path coefficient of the direct effect of professional identity between perceived organizational support and emotional labor is 0.031 and the upper and lower limits of the confidence interval are−0.027 and 0.112, respectively, including zero. This means that there is no direct effect. The total effect path coefficient of professional identity on the relationship between perceived organizational support and emotional labor was 0.111 and the upper and lower limits of confidence interval were 0.038 and 0.193, respectively, excluding zero. We can therefore conclude that professional identity has an absolute mediating effect on the relationship between perceived organizational support and emotional labor.

## Research Conclusions and Implications

### Research Conclusion

During the national pandemic aid operation to Hubei, medical personnel represented the main force of the hospital organization. The main goal of the aid operation was to control the outbreak and successfully offer aid to Hubei as soon as possible. Realizing the maximum value of medical personnel was an important part of the success of the pandemic aid operation. By considering the emotional labor of medical workers in Hubei Province as the breakthrough point and taking professional identity as the intermediary, this study examined the effect of perceived organizational support on the emotional labor of medical workers in Hubei Province. We constructed a theoretical framework with professional identity as the mediating variable and conducted a questionnaire survey on a sample of 170 medical personnel in Hubei Province. We used the empirical method to verify the hypotheses and obtained the following conclusions:

(1) A strong sense of organizational support will encourage medical personnel to invest more emotional labor.
(2) A strong sense of organizational support will promote a stronger professional identity among medical personnel.
(3) A strong professional identity is helpful in encouraging medical workers to invest more emotional labor.
(4) Professional identity completely mediates the influence of perceived organizational support on emotional labor.

### Research Implications

This study found that medical personnel in Hubei felt great support from the hospitals they were working at and that they would show more recognition and understanding of their own work. They were also willing to work hard to achieve the requirements and goals of the hospital and link the achievement of self-goals with the achievement of hospital goals. This means that there is a great sense of organizational support among medical workers in Hubei Province. This psychological perception, enhanced by professional identity, encouraged medical personnel to fight harder in the front lines of pandemic prevention, invest more emotional labor to cure COVID-19 infected patients, and finally defeat the pandemic. Based on our results, in modern hospital management, managers should enhance workers' sense of organizational support and their professional identity. This would enable medical personnel to combine their own career goals with organizational goals. Further, in the event of public emergencies, such as the COVID-19 pandemic, they are willing to invest more emotional labor to achieve organizational goals.

## Data Availability Statement

The original contributions generated for the study are included in the article/supplementary material, further inquiries can be directed to the corresponding authors.

## Ethics Statement

Written informed consent was obtained before the experiments, and the study was approved by the committee of the ethics board of Nanjing University of Chinese Medicine and the latest revision of the Declaration of Helsinki.

## Author Contributions

ZZ and SG designed the study. XW, HB, SY, and YL performed the experiments. XW, GX, and HB analyzed the data. ZZ, GX, and SG wrote the paper. All authors contributed to the article and approved the submitted version.

## Conflict of Interest

The authors declare that the research was conducted in the absence of any commercial or financial relationships that could be construed as a potential conflict of interest.

## References

[B1] Ashforth B. E. Humphrey R. H. (1993). Emotional labor in service roles: the influence of identity. Acad. Manag. Rev. 18, 88–115. 10.5465/amr.1993.3997508

[B2] Back C.-Y. Hyun D. S. Jeung D. Y. Chang S. J. (2020). Mediating effects of burnout in the association between emotional labor and turnover intention in Korean clinical nurses. Saf. Health Work 11, 88–96. 10.1016/j.shaw.2020.01.002 32206378 PMC7078559

[B3] Blau G. J. (1988). Further exploring the meaning and measurement of career commitment. J. Vocat. Behav. 32, 284–297. 10.1016/0001-8791(88)90020-6

[B4] China News Network (2020). National Health Commission of China. Available online at: https://www.chinanews.com/gn/2020/04-07/9149952.shtml (accessed January 01, 2021).

[B5] Delgado C. Upton D. Ranse K. Furness T. Foster K. (2017). Nurses' resilience and the emotional labour of nursing work: an integrative review of empirical literature. Int. J. Nurs. Stud. 70, 71–88. 10.1016/j.ijnurstu.2017.02.008 28235694

[B6] Di Monte C. Monaco S. Mariani R. Di Trani M. (2020). From resilience to burnout: psychological features of Italian general practitioners during COVID-19 emergency. Front. Psychol. 11:567201. 10.3389/fpsyg.2020.567201 33132972 PMC7566043

[B7] Di Trani M. Mariani R. Ferri R. De Berardinis D. Frigo M. G. (2021). From resilience to burnout in healthcare workers during the COVID-19 emergency: the role of the ability to tolerate uncertainty. Front. Psychol. 12:646435. 10.3389/fpsyg.2021.646435 33935905 PMC8085585

[B8] Diefendorff J. M. Erickson R. J. Grandey A. A. Dahling J. J. (2011). Emotional display rules as work unit norms: a multilevel analysis of emotional labor among nurses. J. Occup. Health Psychol. 16, 170–186. 10.1037/a0021725 21244168

[B9] Eisenberger R. Huntington R. Hutchison S. Sowa D. (1986). Perceived organizational support. J. Appl. Psychol. 71, 500–507. 10.1037/0021-9010.71.3.500

[B10] Forouzadeh M. Kiani M. Bazmi S. (2018). Professionalism and its role in the formation of medical professional identity. Front. Psychol. 32:130. 10.14196/mjiri.32.130 30815425 PMC6387805

[B11] Galletta M. Portoghese I. Penna M. P. Battistelli A. Saiani L. (2011). Turnover intention among Italian nurses: the moderating roles of supervisor support and organizational support. Nurs. Health Sci. 13, 185–191. 10.1111/j.1442-2018.2011.00596.x 21595810

[B12] Glomb T. M. Tews M. J. (2004). Emotional labor: a conceptualization and scale development. J. Vocat. Behav. 64, 1–23. 10.1016/S0001-8791(03)00038-1

[B13] Grandey A. A. (2000). Emotional regulation in the workplace: a new way to conceptualize emotional labor. J. Occup. Health Psychol. 5:95. 10.1037/1076-8998.5.1.95 10658889

[B14] Henderson A. C. Borry E. L. (2020). The emotional burdens of public service: rules, trust, and emotional labour in emergency medical services. Public Money Manag. 40, 1–10. 10.1080/09540962.2020.1831180

[B15] Hong Z. Tiao-tiao L. Cai-yun Z. (2010). Testing for reliability and validity of chinese version of the nurse's career identity scale. Chin. Nurs. Manag. 10, 49–51. 10.3969/j.issn.1672-1756.2010.11.018

[B16] Kyratsis Y. Atun R. Phillips N. Tracey P. George G. (2017). Health systems in transition: professional identity work in the context of shifting institutional logics. Acad. Manag. J. 60, 610–641. 10.5465/amj.2013.0684

[B17] Labrague L. J. McEnroe P. D. M. Leocadio MC Van Bogaert P. Tsaras K. (2018). Perceptions of organizational support and its impact on nurses' job outcomes. Nurs. Forum 5, 339–347. 10.1111/nuf.12260 29693264

[B18] Larson E. B. Yao X. (2005). Clinical empathy as emotional labor in the patient–physician relationship. JAMA 239, 1100–1106. 10.1001/jama.293.9.1100 15741532

[B19] Li Y. Liang F. Xu Q. Gu S. Wang Y. Li Y.. (2021). Social support, attachment closeness, and self-esteem affect depression in international students in China. Front. Psychol. 12:399. 10.3389/fpsyg.2021.618105 33746837 PMC7969666

[B20] Ling W. Yang H. Fang L. (2006). Perceived organizational support (POS) of the employees. Acta Psychol. Sinica 38, 281–287. 10.1186/xlxb.0.2006-02-014

[B21] MacMillan R. (1997). Customer Satisfaction and Organizational Support for Service Providers (Doctoral dissertation). Gainesville, FL: University of Florida.

[B22] Matthews R. Smith-Han K. Nicholson H. (2020). From physiotherapy to the army: negotiating previously developed professional identities in mature medical students. Adv. Health Sci. Educ. Theory Pract. 25, 607–627. 10.1007/s10459-019-09942-0 31701305

[B23] Moore M. Hofman J. E. (1998). Professional identity in institutions of higher learning in Israel. Higher Educ. 17, 69–79. 10.1007/BF00130900

[B24] Poghosyan L. Ghaffari A. Liu J. McHugh M. D. (2020). Organizational support for nurse practitioners in primary care and workforce outcomes. Nurs. Res. 69, 280–288. 10.1097/NNR.0000000000000425 32058457 PMC9869754

[B25] Rhoades L. Eisenberger R. (2002). Perceived organizational support: a review of the literature. J. Appl. Psychol. 87, 698–714. 10.1037/0021-9010.87.4.698 12184574

[B26] Russell H. A. (1983). The Managed Heart: The Commercialization of Human Feeling. London: University of California Press, Ltd. p. 223–241.

[B27] Selma S. Selma D. (2015). Effects of the professional identity development programme on the professional identity, job satisfaction and burnout levels of nurses: a pilot study. Int. J. Nurs. Pract. 21, 847–857. 10.1111/ijn.12330 24779558

[B28] Wang J. Liu W. Mi J. Xiao M. Zhao Q. (2020). Status and correlation analysis of perceived organizational support, psychological capital and work engagement among frontline nurses fighting against COVID-19 in Chongqing. Chin. Nurs. Res. 34, 3068–3073. 10.12102/j.issn.1009-6493.2020.17.011

[B29] Willetts G. Garvey L. (2020). Constructing nurses' professional identity through group performance. Int. J. Nurs. Pract. 26:e12849. 10.1111/ijn.12849 32568469

[B30] Yang T. Ma T. Liu P. Liu Y. Chen Q. Guo Y.. (2019). Perceived social support and presenteeism among healthcare workers in China: the mediating role of organizational commitment. Environ. Health Prev. Med. 24:55. 10.1186/s12199-019-0814-8 31481032 PMC6724257

[B31] Zeng Z. Shen J. (2013). Review and prospect of research on emotion studies in organizations. Manag. Rev. Soc. Sci. 2, 73–77. 10.1186/SHGP.0.2013-02-010

[B32] Zhao H. Xi Y. (2017). Emotional labor and turnover intention: emotional exhaustion as mediator and perceived organizational support as moderator. Res. Econ. Manag. 38, 80–86. 10.13502/j.cnki.issn1000-7636.2017.02.009

[B33] Zhou H. Guo Y. (2006). Theory and research of ego identity. Adv. Psychol. Sci. 14, 133–137. 10.3969/j.issn.1671-3710.2006.01.021

